# Hand transplantation: can we balance the risks and benefits?

**DOI:** 10.1177/17531934221132665

**Published:** 2023-01-13

**Authors:** Simon P. J. Kay, David A. Leonard

**Affiliations:** 1 Hand Transplant UK, Leeds Teaching Hospitals Trust, Leeds, UK; 2 School of Medicine, Dentistry & Nursing, University of Glasgow, Glasgow, UK

**Keywords:** Hand transplant, upper limb transplant, vascularized composite allotransplant, risk, benefit

## Abstract

Asking ‘can we balance the risks and benefits?’ implies that a quantification of both risk and benefit in hand transplantation (here the terms hand transplant and hand transplantation refer to allotransplantation of the human hand or hand and part or all of the upper limb or limbs) is possible. Despite all we have learned in recent years about hand transplantation, much remains unknown. Even if reliable methods for quantification of risk and benefit were available, fundamental issues relating to effective communication across the gulf of lived experience between the (presumably) handed surgeon and the handless patient remain. Inherent complexities mean some consider hand transplantation an unsolved problem, but we believe the medical and technical considerations fall within the ambit of a competent multidisciplinary team, and that psychosocial and ethical challenges are open to management through robust frameworks for assessment and decision making, underpinned by an extended period of assessment and dialogue, with candid acknowledgement where uncertainty remains. This respects the patient’s autonomy while addressing the need for a prolonged period of informing consent.

**Level of evidence:** V

## Introduction

The title of this article implies a belief that a quantification of both risk and benefit in hand transplantation (here the terms hand transplant and hand transplantation refer to allotransplantation of the human hand or hand and part or all of the upper limb or limbs) is possible. In leading our multidisciplinary clinics, one of us (SK) likes to remind patients and colleagues of a large, imaginary bin in the corner of the room labelled ‘Don't Know’ in which at least half the questions we ask ourselves, and which we are asked, belong. Even if we had more consistent methods of assessing risk and benefit, there are difficulties at the heart of the matter, in that we may be able to quantify at least approximately the level of risk, but cannot be sure of how to communicate it, while surgeons by definition of the circumstances cannot know the experience of being handless, or one handed, and so cannot estimate the perceived benefit of restitution for a handless person.

Informed consent is an ideal we aim for, and for many procedures surgeons exchange information about risks and the chances of success. However, they rarely assess how that information is interpreted by the patient, nor do they usually assess the cognitive ability of their patients, nor the retention of information over time. We are very often incurious as to how the patient has reached the conclusion to proceed (Boyd, 2015).

Our opinions here are based upon a 12-year experience (10 years since our first transplant procedure), encompassing 14 upper limb transplants in eight patients. During that time, we have declined to operate on over 50 cases referred, advising them for many different and individual reasons that we consider hand transplant unwise or unsafe for them. Of those we have transplanted, we have never advocated hand transplant, but advised that the procedure is well indicated and appropriate but fraught with risks, only some of which we can quantify. This becomes a conversation within the team, which includes the patient, and that conversation continues even after the patient accepts the offer of hand transplant. Finally, it is erroneous to consider the 50 or so patients not transplanted as untreated. The processes of assessment, prosthetic review, and therapy and advice in our multi-disciplinary team (MDT) is exhaustive and many patients benefit, as they testify themselves: it seems the assessment itself has a therapeutic value.

While the inherent complexities may lead some to consider hand transplantation an unsolved problem, we find the medical and technical aspects to be within the grasp of a competent MDT, and the psychosocial and ethical challenges amenable to robust frameworks for assessment and decision making, underpinned by open acknowledgement and communication where uncertainty remains.

## What creates risk in hand transplant and how can this be mitigated?

Surgery entails some risk of adverse outcomes. Hand transplant adds the exceptional complexities of immunosuppression, but also of bringing together tissues from two distinct individuals, and success relies upon congruence between the donor and recipient parts in the disposition of skin incisions, of osteosynthesis and of muscle repairs (matching lengths carefully), but also psychological acceptance of the limb(s) by the recipient (Petruzzo et al., 2020).

The donor and recipient are carefully matched so that blood type and human leukocyte antigen (HLA) profiles in the donor do not encounter existing antibodies in the recipient, which would risk hyperacute rejection (occurring almost immediately on restoration of blood flow). Such antibodies against donor HLA are referred to as donor specific antibodies (DSA) and represent a sensitivity on the part of the recipient to other human antigens. These may have been encountered during previous transfusions, during pregnancies or rarely in other organ or tissue transfers. In our service the recipient is repeatedly assessed over the period of a year for the levels of these DSAs (which may fluctuate), and should they reach a predetermined threshold concentration that recipient cannot accept organs from a donor with the corresponding antigens (Clark et al., 2020).

While no one cares much what their kidney looks like, the appearance of the transplanted limbs is important to the recipient (Kumnig et al., 2022). Often forgotten in the litany of mechanistic risks are the psychological risks after transplantation. These were seen at the most extreme in the first modern era hand transplant, in Lyon in 1998, when the patient repudiated the transferred limb, became uncompliant with immunosuppression, and lost the limb (McIntyre, 2001). In our service a Consultant Clinical Psychologist repeatedly interviews the patient over at least a year to establish what they will accept in terms of limb appearance, and to assess their understanding of the information given, their ability to retain that information and the stability (consistency) of their expectations and demands. These interviews coincide with the blood sampling to assess DSA levels, and the year during which these two assessments take place (subsequent to having been accepted in principle for hand transplant) is known in our service as the ‘year of waiting’. This concept has become a cornerstone of our attempts to mitigate the risks of immunological and psychological rejection, to exchange information essential to consent, and to confirm the stability and understanding of the recipient.

Risks in hand transplant at the time of surgery include the transmission of virus, and the effects of injury itself. Any risk of viral transfer (especially human immunodeficiency virus (HIV), Hepatitis C, Epstein-Barr virus (EBV) and cytomegalovirus (CMV)) is either obviated by matching processes or managed by pre-emptive antivirals (CMV mismatch for example).

In hand transplant we look to assess the risk that the surgery itself poses, and then the risks of the lifelong immunosuppression needed to avoid rejection. In a competent environment the risk to life of the surgery itself for a bilateral transplant at wrist level is small. However, bilateral hand transplant above elbow (or most recently, at the shoulder level) may incur a greater risk due to the sheer volume of tissue transferred, and the consequent reperfusion injury as vascular clamps are removed. While there are strategies to mitigate this, it is our practice to include death from hypotension or metabolic catastrophe as an acknowledged risk in larger volume transplants.

**Figure 1.**
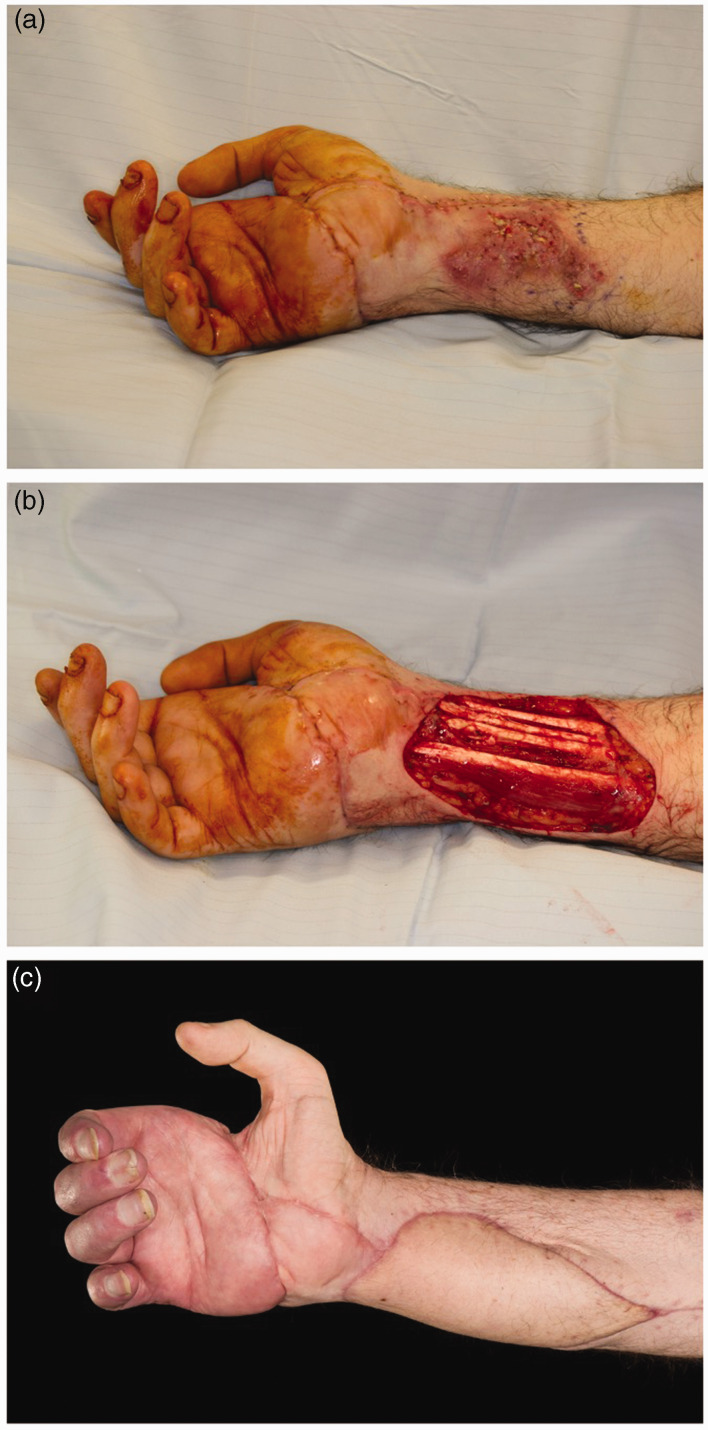
Mycotic infection following upper limb transplantation. (a) Initial presentation with mucormycosis. (b) Post-debridement and (c) Three months after free-flap reconstruction.

If the recipient comes through surgery with the transplants intact and perfused, they then face a myriad of early postoperative risks related to tissue healing (especially bone union and wound closure) and to infection. Such infections may include opportunistic fungal infections, which can be severe and risk fatality (Figure 1).

When healing is complete, regeneration of nerves and return of function is the next milestone. Tacrolimus increases myelinated fibre count, enhances the speed of advancement of the axonal growth cones, and the rapidity of reinnervation can be astounding (Zuo et al., 2020). However, the recovery of extrinsic muscle function depends on whether the native forearm muscles have been conserved or replaced. In the latter case it has been our practice to maintain as great a length of native forearm skeleton as possible, lest the transplant fail and a below elbow prosthesis is then required. This policy has compromised the function of the hand transplant since it has required elevation of donor flexors and extensors from the donor skeleton and reattachment to the native radius and ulna, a manoeuvre that markedly reduces muscle power and function.

Finally, we come to the longest lasting concern in terms of risk, and that is the effect of immunosuppression. This is the risk that most opponents of the procedure home in upon, and it requires some interpretation.

Immunosuppression generally starts with administration of an induction agent to deplete lymphocytes immediately before transplant, followed by administration of other (oral) agents to maintain immunosuppression via a range of mechanisms (Rifkin et al., 2021). In our programme, induction is achieved with a single dose of alemtuzumab, a monoclonal antibody against the CD52 antigen present on mature lymphocytes. Others have used polyclonal agents (anti-thymocyte globulin; ATG) to similar, albeit more widely targeted effect. Maintenance therapy typically consists of three daily oral medications: corticosteroids, mycophenolate mofetil (MMF) and tacrolimus, each of which contributes to control of rejection, but brings its own risks and side effects.

Corticosteroids (in doses that are rapidly tailed to around 7.5 to 10 mgs prednisolone a day), are an almost universal component of immunosuppression and act via numerous pathways to inhibit proinflammatory transcription factors and promote anti-inflammatory genes. Side effects may include raised blood pressure (treatable) and impaired glucose tolerance (also treatable). They may also encourage weight gain, or steroid facies (rarely persistent), and may contribute vulnerability to opportunistic infections.

MMF is a prodrug of mycophenolic acid, which inhibits proliferation of T- and B-lymphocytes and impairs recruitment of lymphocytes and monocytes to sites of inflammation. MMF exerts broad anti-inflammatory activity, with actions against mechanisms of both acute and chronic rejection. It is generally well tolerated but may confer dose-related bowel symptoms and myelosuppression, as well as other less specific drug side effects.

Finally, tacrolimus is a mainstay of immunosuppression. Acting via inhibition of calcineurin to reduce transcription of interleukin-2 (IL-2) and other inflammatory mediators, it is an effective inhibitor of key components in the cell-mediated immune response, but also has a narrow therapeutic dose range and a plethora of recorded side effects. Notable among these is nephrotoxicity, and particularly in patients who have suffered quadrimembral limb loss from sepsis (and who often exhibit impaired renal function) this can lead to dependency on renal support, and in the longer term for renal transplantation. This effect can be mitigated by switching to other agents once healing is complete (e.g. sirolimus, which is less nephrotoxic but a powerful inhibitor of healing). However, tacrolimus is so effective that many patients with solid organ transplants are maintained on it for life, and some will suffer renal impairment as a result.

Having seen that immunosuppression can result in metabolic and organ dysfunction as well as impaired response to infection, we should also consider that it can result in an increased rate of malignancy (Geissler, 2015). The malignancies that show increased rates are especially skin cancers, and those related to viral aetiology, including lymphoproliferative disorders (hence the importance of viral status matching, particularly for EBV) (Zafar et al., 2008). However solid organ tumours, such as prostate, bowel and breast malignancies, may have a relatively small increase in risk, perhaps double. Since (despite public perception) they are themselves relatively unusual, the likelihood of developing one as a result of immunosuppression has been likened to increasing the chances of winning a lottery by buying two tickets instead of one (personal communication Dr Richard Baker, Consultant Transplant Physician, 2012).

From these considerations of the adverse effects of immunosuppression it may be expected that treatment sufficient to suppress rejection will have consequences over a lifetime, and inevitably lead to shortening of a life over that expected of a healthy individual of the age at which they receive their transplant.

It has been suggested that it is unjustified to give such powerful medication to healthy individuals, especially for a non-life saving procedure; however, it should be acknowledged that our information about the adverse effects of such medication comes from those with solid organ transplants (SOT), who are already unwell, as evidenced by their need for organ replacement. These suggestions embody a number of assumptions, the first being that people with upper limb loss are healthy. We do not know the natural history of handlessness, but intuitively it seems likely that such people will be disadvantaged in employment, and so socio-economically, and perhaps therefore in general health (Mair and Jani, 2020). It is likely they will suffer psychosocial consequences of their deformity and may fall into adverse behaviours, including substance abuses. It is established that all these factors have a deleterious effect on life expectancy (Whiteford et al., 2013). So, while a hand transplant may not be immediately life preserving, it may reasonably be hypothesized that it confers benefits that mitigate the likely life-shortening effects of handlessness, even when the consequences of immunosuppression are considered.

It is worth considering that some subsets of SOT are not systemically unwell (for instance those who have lost renal function through single nephrotoxin exposure) and it may be possible to extrapolate from these cases to divine some measure of the life shortening effect of immunosuppression. This might be thought of as a proportion of natural remaining years, and if so would have more consequences for the younger patient, who paradoxically is likely to get greater and longer value from hand transplant than older patients, an important consideration when advising about risk.

## Communicating and understanding risk and understanding ‘understanding’

When we discuss risk, we should clarify what the patient is risking, whether it be risk of death, or of prolonged incapacity, or poor outcome or perhaps pernicious long-term consequences, including early or eventual loss of the new limbs.

When trying to convey risk to a patient, the quantity (Q) of risk is a product of the likelihood of it occurring (frequency, F) and the extent of the consequences if that risk does materialize (C): (Q = C × F). Of course, we cannot assign numerical values to these concepts. Thus, to get a risk into perspective we often rely on analogy: a common bacterial infection after operation has a high frequency, but is easily treated and so is of low consequence and might be expected not to influence the decision greatly. A serious consequence of a rare occurrence (e.g. Post Transplant Lymphoproliferative Disorder; PTLD) might have a greater influence on a patient’s decision, but the low value of F may be of some comfort, especially if a screening system were in place to detect the consequence.

These analogies and examples may seem clear to readers, but it should be borne in mind that many patients are less able to make such analyses. For this reason, as part of our ‘year of waiting’, we assess the ability of patients to undertake abstract reasoning of this sort, and their ability to understand. We then employ measures not only to communicate in terms that they can comprehend, but also to check that they have understood the concepts, can put them into their own words and reiterate them in an enduring fashion. Doing this has given us an appreciation of how fleeting and inadequate most so-called informed consent processes are. Despite our best efforts, it is probably true that no physician can understand how well a patient understands risk; in other words, we cannot live their experience nor empathize with their decision making, influenced as it is by so many historic, internal and external actors. We make a best attempt and comfort ourselves that it is a good enough attempt, while probably many doctors are more concerned to be able to demonstrate, if ever necessary, that they have complied with a doctrine of informed consent.

### The benefits of hand transplantation

Following a somewhat nihilistic view of the understanding of risk, and of sharing that with our patients, it may not be a surprise to find that we also believe it is very difficult to quantify the benefits of hand transplant. This is not because there are none, but because there are so many, whose ranking is not immediately obvious, and because, to understand the benefits of hand transplant, first we must understand the state of handlessness. It seems likely that no surgeon currently practising has been handless, and so no surgeon currently practising can fully appreciate that experience. (Although Harvey Cushing memorably remarked that he looked forward to the day a surgeon who had no hands were to be appointed, since operating is the least part of the job.)

When surgical services present their outcomes from hand transplant, we usually see charts of grip strength, range of movement, sensory function and composite score, such as the Disabilities of Arm, Shoulder & Hand (DASH) or Hand Transplantation Score System (HTSS). While these uniformly demonstrate benefit, they reduce the upper limb to a sensate mechanical appendage, ignoring its human qualities and the behaviours it empowers (Wells et al., 2022). These include its role in communication (passive and active), the caring nature of touch or hand holding, its role in sex, and its ability to interrogate, and direct our responses to, our environment. The comparison of the hand with upper limb prosthetics has encouraged a reductionist view, but an upper limb prosthesis confers only movement and some power, while a transplanted upper limb should confer silent movement, power, sensibility, warmth to touch, sweating and a natural human appearance. Further, it is always attached, never needs a battery charge, and repairs itself (healing). Almost none of these qualities are considered in the outcome data most often presented.

A further quality of transplantation has been drawn to our attention by a patient. He talked about feeling ‘complete at last’ after a unilateral hand transplant at the level of the mid humerus, even though function was limited. He felt, and continues to feel, that this was a very important quality for him, and was distinct from aesthetics (which is a quality appreciated by the external observer) and related more to his internal perception of himself and of his hands. Just as we live behind our face, which senses and interacts with the world (taste, sight, touch, kissing, eating, mastication, speech and facial expression) we do the same with our upper limbs, which act like an extension of our face, but also co-operate before us, one with the other and both with the face to form a ring in which we live and which is constantly incomplete if one or both hands is absent. This is an outcome from hand transplant that is just as incalculable as others.

Reverting to the measurable rather than the abstract, the mechanical and sensory ability of a hand transplant is related to the level of transection, and the damage, in the native part. Of our 14 transplants in eight patients (at the time of writing), one patient (referred to above) had an undiagnosed brachial plexus injury discovered at the time of transplantation, hence the functional result was disappointing (but still useful and welcome). In contrast, another patient who had a mid-humeral transplant had a surprisingly good result. After 14 hand transplants we are beginning to be able to predict the likely musculoskeletal results quite accurately, and the depth of our psychology assessments, shared with the extended team, allow us to anticipate many aspects of the more global behavioural outcome.

### Addressing the unknown

A comprehensive review of current hand transplantation research goes well beyond the scope of this article, but it would be remiss not to acknowledge the broad spectrum of work being undertaken to advance our understanding of the fundamental immunological processes at play, and the factors influencing risk and clinical outcomes. The range of relevant basic science and translational inquiry is extensive, including topical and targeted immunosuppression delivery (Feturi et al., 2022), ex-vivo perfusion (Amin et al., 2021), graft modification to reduce immunogenicity (Lei et al., 2021) and the continued effort to characterize the mechanisms of immune recognition and response, with the ultimate aim of unlocking the door to transplantation without the need for lifelong immunosuppression (transplant tolerance) (Leonard et al., 2021).

These efforts are of critical importance, but since both introduction of major new advances, and assessment of outcomes within current clinical practice, ultimately rely upon statistically valid comparisons there is also a pressing need for robust reporting. So, while the number of cases undertaken by any one unit remains relatively small, achieving a statistically robust dataset will necessitate greater collaboration between units. To this end we believe the most pressing priorities for research in this field are commitments to open and accurate reporting of clinical outcomes and complications, including submission of cases to the International Registry (handregistry.com), and agreement of a minimum dataset for inclusion in such reports.

## Conclusion

One thing that hand transplantation has impressed on our whole team in the 10 years since our first case is that the undertaking is complex and achieving results relies upon the co-operation of a large number of expert disciplines. Taken together with the individual nature of each case, this means there is no simple formula for assessing risk, or benefit. That does not mean we cannot give our patients some of the information they need, but always we are aware of that large bin marked ‘Don't Know’ that sits by our side. Being candid about our lack of knowledge, and candid about our ‘best guess’ approach is only fair, but in the end, faced with a year of contact and facts and opinions, our patients will always make a decision based on their heritage, their lives, their experience and their emotions. That we cannot impose a rigid scientific and numerical structure on that decision should be recognized and embraced. Few of us carry a spreadsheet of risk in our minds as we navigate the many complex decisions in life, and indeed many decisions are made before we consider them analytically. Hand transplant may be no different. We must continue to evaluate our outcomes, to communicate everything we know and to explain why some things are unknown and possibly unknowable.
